# Pesticide pollution in freshwater paves the way for schistosomiasis transmission

**DOI:** 10.1038/s41598-020-60654-7

**Published:** 2020-02-27

**Authors:** Jeremias M. Becker, Akbar A. Ganatra, Faith Kandie, Lina Mühlbauer, Jörg Ahlheim, Werner Brack, Baldwyn Torto, Eric L. Agola, Francis McOdimba, Henner Hollert, Ulrike Fillinger, Matthias Liess

**Affiliations:** 1 Helmholtz Centre for Environmental Research − UFZ, Department System-Ecotoxicology, Permoserstrasse 15, 04318 Leipzig, Germany; 2 RWTH Aachen University, Department of Ecosystem Analysis, Institute for Environmental Research, Worringerweg 1, 52074 Aachen, Germany; 3 International Centre of Insect Physiology and Ecology (icipe), Human Health department, P.O. Box 30772-00100, Nairobi, Kenya; 4 Egerton University, Biological sciences, P.O Box 536-20115, Njoro, Kenya; 5 Ruprecht-Karl-University of Heidelberg, Faculty of Biosciences, Im Neuenheimer Feld 234, 69120 Heidelberg, Germany; 6 Centre for Biotechnology Research and Development, Kenya Medical Research institute (KEMRI), P.O. Box 54840-00200, Nairobi, Kenya; 7 The Technical University of Kenya, P.O. Box 52428-00200, Nairobi, Kenya; 8 Department Evolutionary Ecology and Environmental Toxicology, Institute of Ecology, Evolution and Diversity, Faculty Biological Sciences, Goethe University Frankfurt, Frankfurt, 60438 Germany

**Keywords:** Agroecology, Freshwater ecology, Epidemiology

## Abstract

Schistosomiasis is a severe neglected tropical disease caused by trematodes and transmitted by freshwater snails. Snails are known to be highly tolerant to agricultural pesticides. However, little attention has been paid to the ecological consequences of pesticide pollution in areas endemic for schistosomiasis, where people live in close contact with non-sanitized freshwaters. In complementary laboratory and field studies on Kenyan inland areas along Lake Victoria, we show that pesticide pollution is a major driver in increasing the occurrence of host snails and thus the risk of schistosomiasis transmission. In the laboratory, snails showed higher insecticide tolerance to commonly found pesticides than associated invertebrates, in particular to the neonicotinoid Imidacloprid and the organophosphate Diazinon. In the field, we demonstrated at 48 sites that snails were present exclusively in habitats characterized by pesticide pollution and eutrophication. Our analysis revealed that insensitive snails dominated over their less tolerant competitors. The study shows for the first time that in the field, pesticide concentrations considered “safe” in environmental risk assessment have indirect effects on human health. Thus we conclude there is a need for rethinking the environmental risk of low pesticide concentrations and of integrating agricultural mitigation measures in the control of schistosomiasis.

## Introduction

Schistosomiasis, also called bilharzia, is among the tropical diseases with the highest impact on socio-economic development, only exceeded by malaria. Approximately 218 million people are infected worldwide^1^. Infection has been strongly associated with long-term disabilities^2^. The number of deaths due to schistosomiasis is poorly documented with estimates ranging between 11,700^3^ to 280,000 each year^4^ because of hidden pathologies such as liver and kidney failure^5^. Schistosomiasis is caused by flatworms of the genus *Schistosoma* which parasitize humans as their definitive host (supporting the adult life stage of the parasite). The intermediate hosts are freshwater snails of the family planorbidae which release infective larval stages (cercariae) into the water. Transmission occurs when humans are exposed to water containing infected host snails; direct infection from person to person is not possible^6^. People are infected during routine agricultural, domestic, occupational and recreational activities, which expose them to infested water. Over 80% of afflicted people live in sub-Saharan Africa^7^, but the disease concerns public health in most (sub)tropical countries worldwide^7^ and has recently established in Europe^8^.

Control strategies against schistosomiasis focus on the treatment with praziquantel that kills the adult worms in the human host. However, even mass drug administration does not prevent re-infection in infested water, and schistosomiasis has been observed to rebound within short time^9^. For the sustainable control of schistosomiasis, it is also essential to interrupt the infection cycle by control of the intermediate hosts^10–12^. Host snails are susceptible to predation by organisms such as shrimps^13^ and ostracods^14^ which have been applied as biological control agents. Additionally, host snails are susceptible to competition from other snails and insects that feed on periphyton (microbes attached to surfaces), detritus and water plants^15–17^. Spreading of schistosomiasis has been often linked to the loss of biodiversity and the ecological degradation of freshwater habitats^10,18,19^. The findings suggest that host snails are significantly controlled by antagonistic species in natural habitats but that this ecosystem service is sensitive to anthropogenic impact. Therefore, it is essential to identify key environmental factors that drive the interactions of host snails with their associated community.

Recently, agricultural pesticides have returned to the focus of public attention as causes for the worldwide decline in insects and biodiversity^20–22^. Tropical regions, characterized by extensive agriculture and heavy rainfalls, are known areas of endemicity of schistosomiasis. In such conditions there is a high risk of surface run-off that washes pesticides from agricultural fields into adjacent freshwater systems^23^. However, information on pesticide concentrations in tropical freshwaters and their effects on the macroinvertebrate community are often fragmented and inadequate^24,25^. In temperate latitudes, snails are known as one of the macroinvertebrate taxa being most tolerant to pesticides^26^. In mesocosms, high concentrations of insecticides and herbicides favoured host snails indirectly through the reduction of predators and through the replacement of suspended algae with periphyton that serves as food for snails^27^. Additionally, even low levels of agricultural pesticides result in a typical replacement of sensitive macroinvertebrates by more tolerant taxa in mesocosms^28^ and in natural streams^29,30^. These effects are usually driven by insecticides that are most toxic to many macroinvertebrates^30^. Therefore, we hypothesized that pesticide pollution may favour highly tolerant snails that host human-pathogenic schistosomes over their more sensitive natural enemies and thus increase the risk of schistosomiasis transmission.

## Results

**Figure 1:**
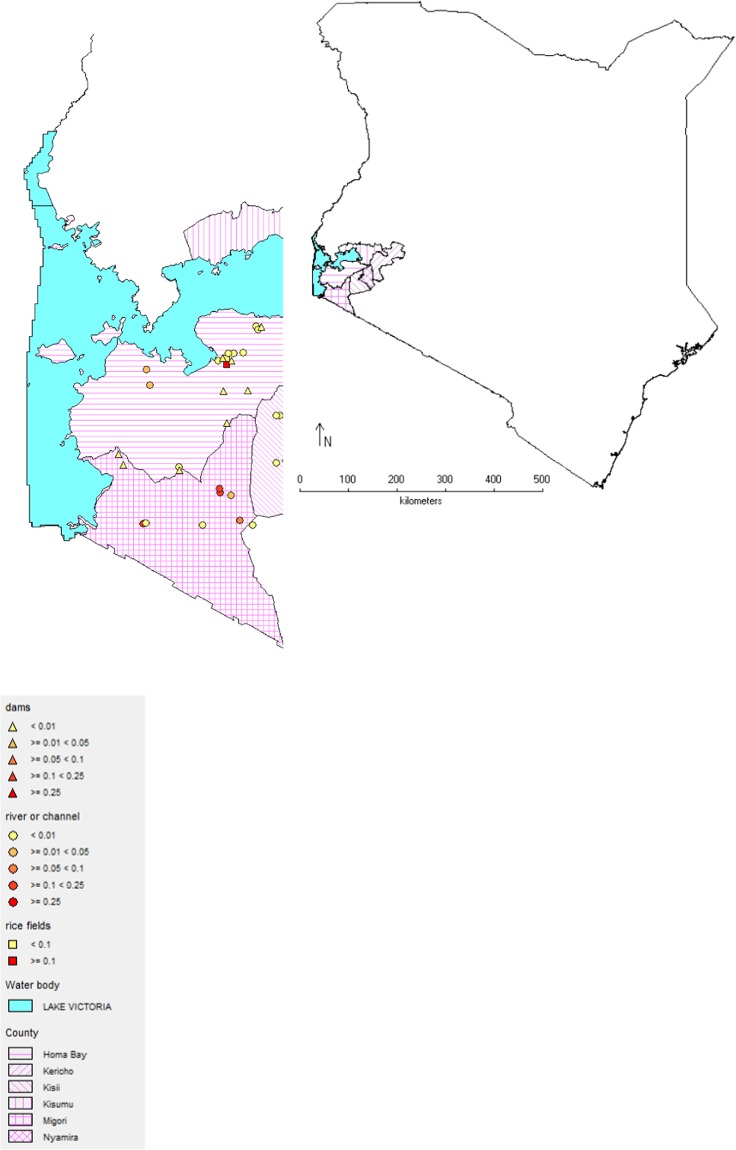
Dominance of host snails of the pathogens of human schistosomiasis among the study sites. Sites were depicted by the shapes of the icons as either reservoirs (triangles), streams/channels (circles) or rice fields (squares). The dominance of host snails (number of transmitting planorbid snails/total number of individuals per site) is represented by the shade of the icon. Maps created using DIVA-GIS 7.5.0. https://diva-gis.org/.

We studied how pesticide pollution and additional environmental factors affect the macroinvertebrate community composition in a typical endemic region of schistosomiasis. For this, we sampled 48 freshwater sites in the Kenyan Lake Victoria Basin (Fig. 1). The habitats ranged from small and medium-sized streams to irrigation channels, oxbow lakes, reservoirs and rice fields, and thus covered the main inland transmission sites in the study area^31^. Each site was monitored once during the rainy season in October 2017. To confirm the hypothesized high pesticide tolerance of host snails, we collected host snails and other common invertebrates and tested their acute sensitivity to two insecticides covering different modes of action.

### Pesticide tolerance of *Schistosoma* host snails

**Figure 2:**
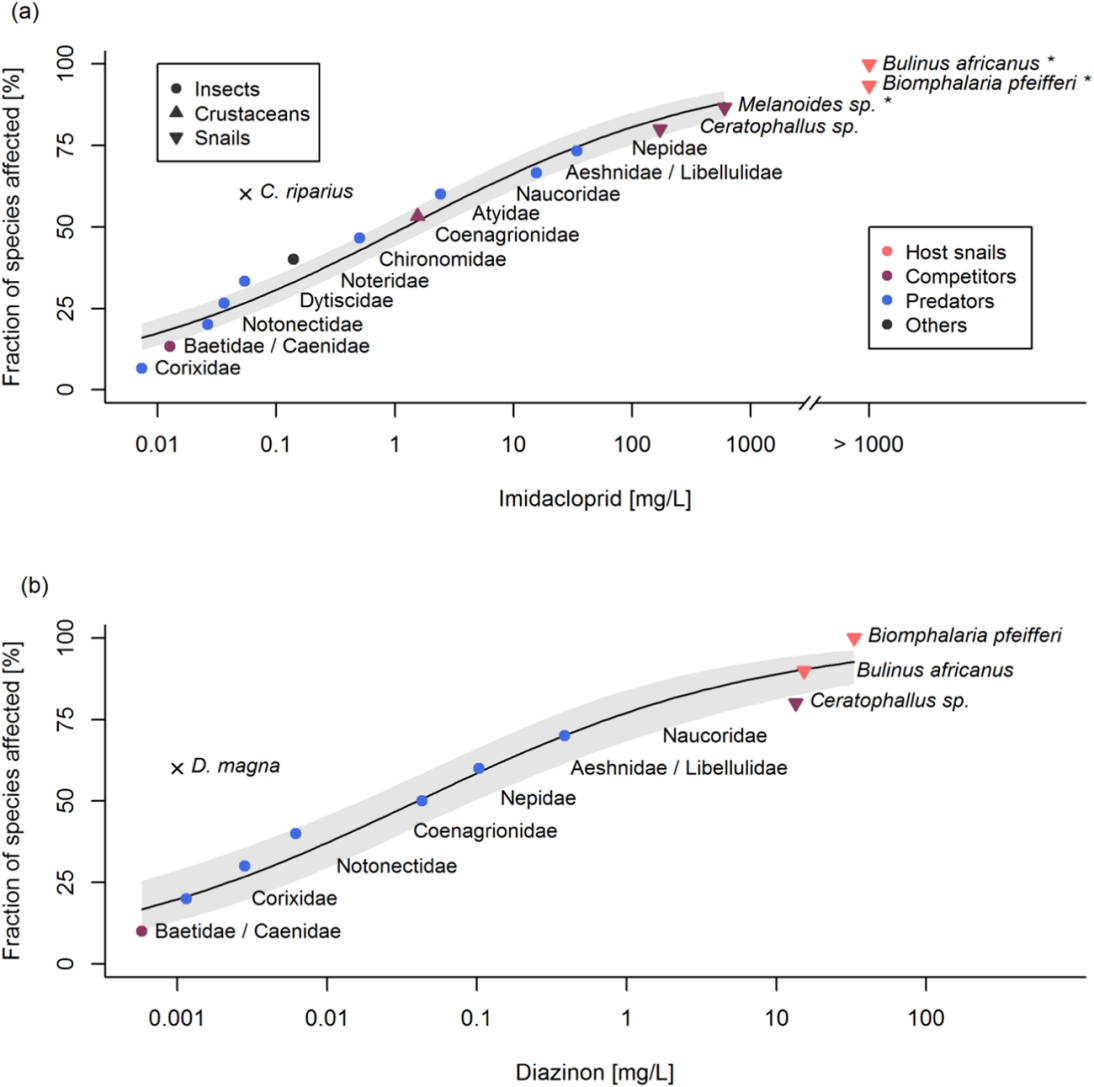
Species sensitivity distribution (SSD) of freshwater macroinvertebrates from the study region to common agricultural insecticides. Data points show the acute LC_5024h_ for various species. The SSD curves were fitted using a quasibinomial GLM with logit-link; means ± 95% confidence intervals are shown. (**a**) Sensitivity distribution to the neonicotinoid insecticide imidacloprid. *χ²* = 230.69, res. df = 11, *p* < 0.001, McKelvey-Zavoina’s pseudo-*R²* = 0.29. For *Melanoides sp*., *Bulinus africanus* and *Biomphalaria pfeifferi* the LC_50_ exceeded the highest test concentration and was extrapolated from non-linear regression (*Melanoides sp*.) or estimated. (**b**) Sensitivity distribution to the organophosphorus insecticide Diazinon. *χ²* = 115.89, res. df = 8, *p* < 0.001, McKelvey-Zavoina’s pseudo-*R²* = 0.40. (**a,b**) The acute LC_5048h_ of the most sensitive standard reference taxa (*Chironomus riparius* and *Daphnia magna*) was added for comparison^32,78^ and used for the calculation of toxic units (see text).

Among all macroinvertebrates tested, host snails of human-pathogenic schistosomes showed the highest tolerance to the neonicotinoid insecticide imidacloprid and to the organophosphorus insecticide diazinon (Fig. 2a,b), both of which were commonly found at the study sites. The acute median lethal concentration of imidacloprid after exposure for 24 h (LC_5024h_, concentration that killed 50% of test organisms) ranged from 0.007 mg/L for corixidae (true bugs) to 599 mg/L for the non-host snail *Melanoides sp*. The mortality of the host snails *Bulinus africanus* and *Biomphalaria pfeifferi* remained below 10% even at the highest concentration tested (165 mg/L) so that we could only estimate their respective LC_50_. This test concentration was close to the solubility limit of imidacloprid in water (610 mg/L^32^), indicating very high insecticide tolerance of the host snails. The LC_5024h_ of diazinon for other taxa ranged from 0.5 µg/L for baetidae and caenidae (mayflies) to 13.5 mg/L for the non-host snail *Ceratophallus sp*. Again, the host snails *B*. *africanus* (15.2 mg/L) and *B*. *pfeifferi* (33.0 mg/L) were the most tolerant species.

### Pesticide pollution in the study area

The surveyed aquatic habitats showed considerable agricultural pesticide pollution. We analyzed 28 commonly applied active substances and degradation products and detected all the compounds in water samples, ranging from 5 to 27 (median = 20) substances per site. To quantify the toxicity, pesticide concentrations were converted to toxic units (TU) using the formula $TU=lo{g}_{10}(\frac{Concentration}{LC{50}_{reference}})$ with *Concentration* being the measured concentration of a pesticide and LC_50*reference*_ being the LC_50_ of that pesticide for a standard reference organism (typically *Daphnia magna*, Table S1 in the Supplementary Material)^33^. Toxic units of −1 or −2 represent pesticide concentrations of 1/10 or 1/100 of the LC_50_, respectively. Toxic units of pesticides in agricultural European, Siberian and Australian streams typically reach from ≤−5 to −1^34,35^.

In two sites all pesticides detected were below the limit of quantification such that the toxic unit of the most toxic compound (TU_max_) could not be calculated. However, in the other sites, the TU_max_ ranged from −6.4 to −1.2 with a median TU_max_ of −2.3 (Table S2 in the Supplementary Material). The most toxic substances found were the organophosphorous and carbamate insecticides bendiocarb (most toxic substance at 17 sites, TU up to −1.66, Table S3 in the Supplementary Material), diazinon (most toxic at 15 sites, TU up to −1.71) and pirimiphos-methyl (most toxic at 5 sites, TU up to −1.21). Due to difficulties in the quantification of very low pesticide concentrations, the minimum TU_max_ was set to −5 for further analyses, a threshold at which typically no ecological effects have been observed in field studies^29,34,35^.

### Environmental factors driving host snail abundance

We investigated the influence of 27 environmental variables on the abundance of host snails, covering habitat type, land use, water chemistry and the composition of the macroinvertebrate community (Table S4 in the Supplementary Material). Host snails were found in 9 out of a total of 48 sites investigated in 2017; at one site they were infected with human-pathogenic schistosomes. The abundance of host snails encompasses the incidence, i.e. the probability of a population to occur at a given site, and the density of existing populations. Both endpoints can be driven by different environmental factors and thus were analyzed separately in a first step.

**Figure 3:**
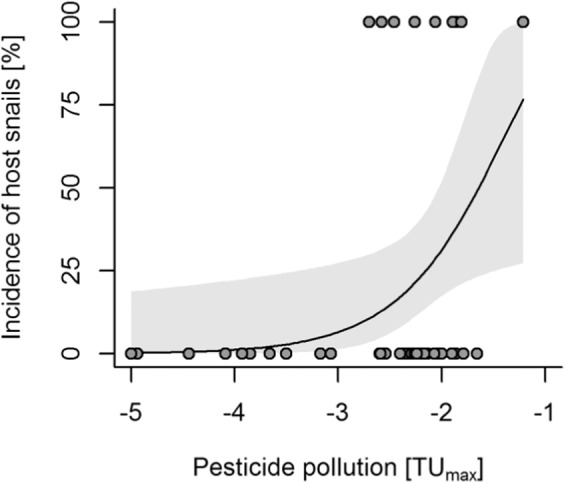
Pesticide pollution increases the incidence (probability of occurrence) of snails that act as hosts of schistosomiasis. Binomial GLM with complementary log-log link function; *χ²* = 7.60, res. df = 46, *p* = 0.006, McFadden’s pseudo-*R²* = 0.16. Means ± 95% confidence intervals are shown. Pesticide pollution was quantified as log_10_ of the maximum ratio of a pesticide concentration measured in a grab sample of water vs. the acute LC_50_of that pesticide for a standard reference organism (TU_max_). TU_max_ of marginally polluted sites (*n* = 4) was set to a minimum of TU −5.

When each environmental variable was considered individually, the incidence of host snails increased significantly with pesticide toxicity (*n* = 48, *χ²* = 7.71, res. df = 46, *p* = 0.005, Fig. 3), species diversity (*χ²* = 4.42, res. df = 46, *p* = 0.035) and species richness (*χ²* = 4.39, res. df= 046, *p* = 0.036). Additionally, the incidence of host snails decreased with increasing dissolved oxygen (*χ²* = 8.06, res. df = 46, *p* = 0.004) and with the increasing dominance (proportion on all macroinvertebrates) of other grazers and herbivores that act as potential competitor species (χ² = 9.09, res. df = 46, *p* = 0.003; Table S4 in the Supplementary Material).

Environmental effects on the density of host snail populations were driven by a single stream (Table S5 in the Supplementary Material). This stream was characterized by extraordinarily high numbers of host snails and other macroinvertebrates. The site was located 100 m downstream of a bathing and washing area and was the only site at which infected host snails were found. When we excluded this site as an outlier, population density was explained only by a decrease in density with increasing turbidity (*n* = 8, *χ²* = 4.50, res. df = 6, *p* = 0.034).

In a second step, we combined the effects identified on the incidence and population density in a hurdle model in order to rank the relevance of the environmental variables in explaining the overall abundance of host snails. Stepwise regression identified that the incidence of host snails increased primarily with pesticide pollution, followed by an increase with the decreasing dominance of potential competitors, with increasing species richness and with a decreasing amount of dissolved oxygen (Table 1). The density of host snail populations only decreased with turbidity.

**Table 1:** Minimal adequate model for environmental effects on the abundance of *Schistosoma* host snails.

Term	Coefficient	Std. error	*z*	*p*	
**Count part (zero-truncated negative binomial with log-link; models population density)**					
Intercept	1.92	0.60	4.58	0.001	**
Turbidity	−3.12	1.17	−2.68	0.007	**
ln(Distribution coefficient)	−0.58	0.79	−0.74	0.461	
**Zero part (binomial with complementary log-log-link; models incidence)**					
Intercept	−5.11	1.90	−2.69	0.007	**
Pesticide pollution	2.73	1.29	2.12	0.034	*
Dominance competitors	−2.30	1.09	−2.12	0.034	*
Species richness	1.81	0.92	1.97	0.048	*
Dissolved oxygen	−0.92	0.45	−2.02	0.044	*

We selected all environmental variables that on their own showed a significant effect on the incidence or on the population density of host snails and combined them in an additive hurdle model. Using backward elimination based on likelihood ratio tests, non-significant environmental variables (species diversity) were removed. Because data have been standardized, importance of the environmental variables on the incidence or on the population density of snails can be compared within each part of the model based on their regression coefficients; coefficients far from zero indicate high (positive or negative) impact. Log-likelihood = −42.43 on 8 df and 40 res. df; McFadden’s pseudo-R² = 0.28.

**Figure 4:**
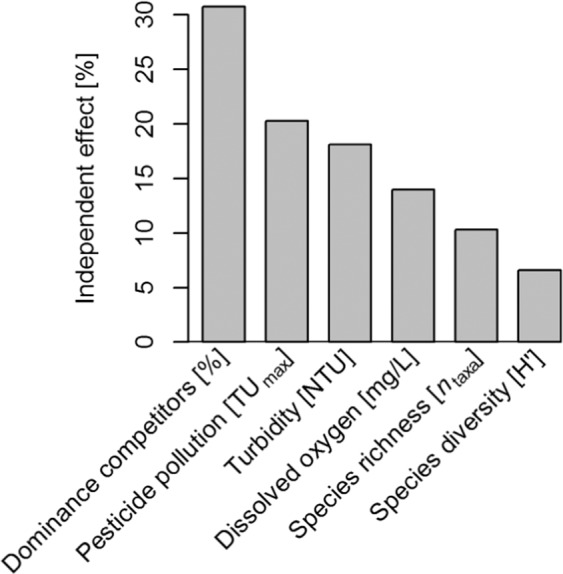
Ranking the relevance of environmental variables in driving the abundance of host snails. We combined all environmental variables that on their own showed a significant effect on the incidence or on the population density of host snails and combined them in a hurdle model. The model was subjected to hierarchical partitioning to identify the independent contribution of each environmental variable to the goodness-of-fit (quantified as log-likelihood of the hurdle model). Each step, an environmental variable was either included both in the zero and the count part of the model or excluded completely.

Results from stepwise regression are sensitive to the method used for model selection. Therefore, we additionally applied a multi-model approach by subjecting the full hurdle model to hierarchical partitioning (Fig. 4). Here the abundance of host snails was most strongly affected by the dominance of potential competitors, followed by the effect of pesticide pollution. In accordance with the results from backward selection, turbidity, species richness and dissolved oxygen showed intermediate effects, and species diversity was least important again.

**Figure 5:**
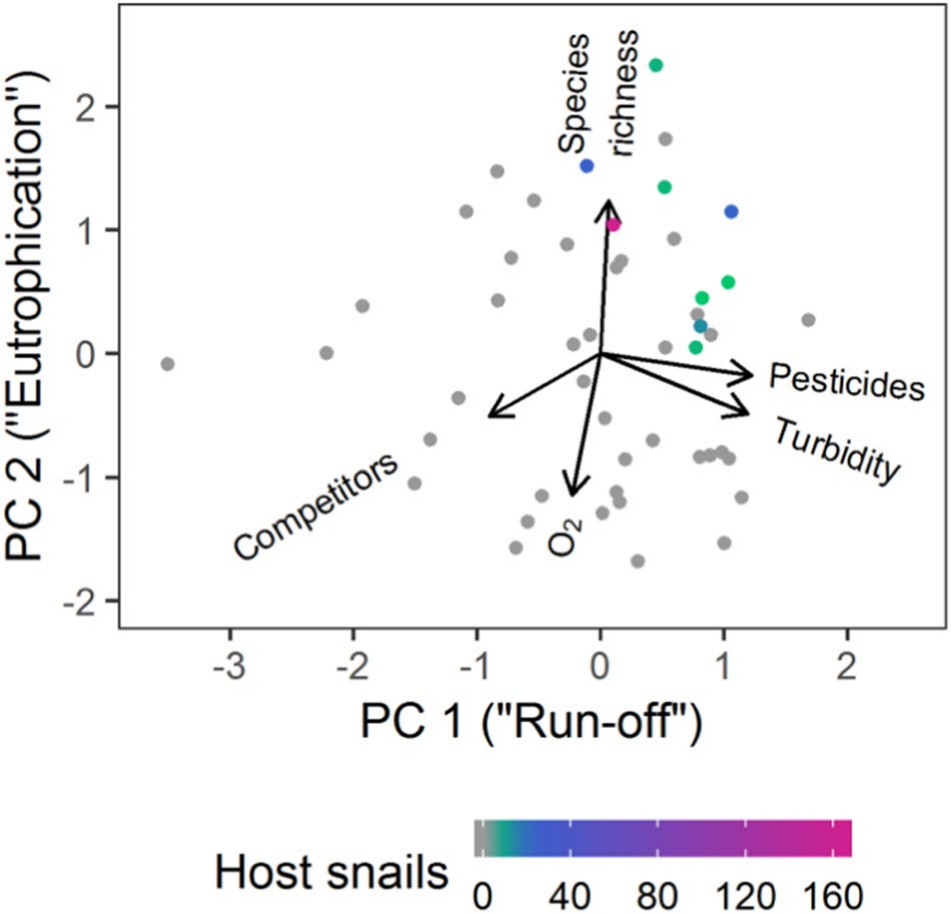
Principal component analysis of the environmental variables that drive the abundance of *Schistosoma* hosts. The 1^st^ principal component explains 33.0% of the variation and is associated with pesticide pollution, turbidity and the dominance of potential competitor species of the host snails. The 2^nd^ principal component explains 29.4% of the variation and is associated with the species richness, dissolved oxygen and again with the dominance of competitors. Colors indicate the number of host snails collected.

In the next step, we confirmed the main drivers that potentially underlie the identified environmental variables using a principal component analysis (PCA). The first principal component explained 33.0% of the total variation among the sites and was associated with typical effects of surface run-off after heavy rainfall (Fig. 5): It increased with pesticide pollution and turbidity and with decreasing dominance of potential competitor species of the host snails. The second principal component additionally explained 29.4% of the variation and increased with species richness, with a decreasing amount of dissolved oxygen (indicating increasing oxygen consumption) and with decreasing dominance of potential competitors. Thus, the second principle component likely reflected an increase of host snails with eutrophication that supports more taxa but results in oxygen depletion. Moreover, the second principal component increased with the overall number of macroinvertebrate individuals as an indicator of productivity (*n* = 48, *F* = 8.12, res. df = 46, *p* = 0.007, *R²* = 0.15) which further supported its interpretation as eutrophication. Host snails were only found when both the effects of run-off and eutrophication were high which resulted in a decreased dominance of potential competitors (Fig. 5).

### Ecological mechanisms

**Figure 6:**
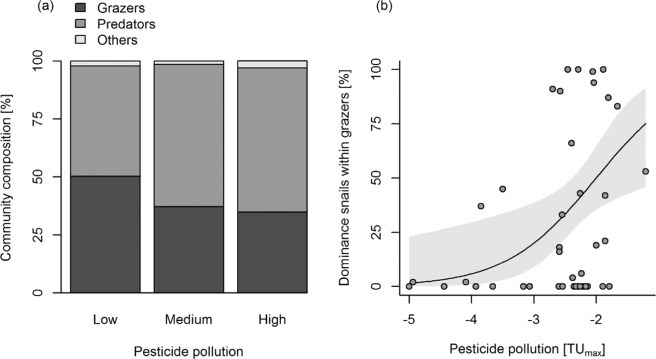
Pesticide pollution favors tolerant snails over less tolerant competitors. (**a**) No significant change in the community composition of grazers, predators and other taxa with pesticide pollution (PERMANOVA; *n* = 48, *F* = 0.80, res. df = 46, *p* = 0.502, *R²* = 0.02). For the graph, the range of TU_max_ values was evenly split in three categories, and for each pollution category the mean proportion of each guild on the macroinvertebrate community is shown. Because some taxa belong to more than one guild, we calculated proportions as the individuals in a guild divided by the summed-up individuals in all guilds (≠ the total individual number) so that the proportions sum up to 1. (**b**) Within the guild of grazers, the dominance of snails increases with pesticide pollution (*n* = 47, *χ²* = 13.82, res. df = 45, *p* < 0.001, McKelvey-Zavoina’s pseudo-*R²* = 0.37). One site was omitted because no grazers were found. Quasi-binomial GLM with logitlink function; means ± 95% confidence intervals are shown.

To better understand the ecological mechanisms through which pesticides affect host snails, we investigated effects of pesticides on the macroinvertebrate community composition. Pesticide pollution affected neither the dominance of all grazers (host snails and their potential competitors; *n* = 48, *χ²* = 0.41, res. df = 46, *p* = 0.520) nor of predators (*χ²* = 0.37, res. df = 46, *p* = 0.541) or other macroinvertebrates (*χ²* = 2.63, res. df = 46, *p* = 0.105). Thus, the overall distribution of grazers, predators and other taxa within the community did not significantly change with pesticide pollution (Fig. 6a). However, within the guild of grazers, pesticide pollution increased the dominance of snails (Fig. 6b) which were much more tolerant to pesticides than their highly sensitive insect competitors (Fig. 2). In contrast, pesticide pollution did not affect the composition of predatory macroinvertebrates (PERMANOVA; *n* = 48, *F* = 0.78, res. df = 46, *p* = 0.679) which generally showed intermediate sensitivity to pesticides (Fig. 2). Additionally, the taxonomic composition of potential predators had no effect on the balance of snails vs. potential competitors: The first principal component of a PCA on the composition of predators did not explain the dominance of snails within the grazers (*n* = 47, *χ²* = 0.02, res. df = 45, *p* = 0.890); the same was observed for higher principal components. Therefore, we conclude that pesticides indirectly favored host snails through negative effects on their competitors but not on their predators.

## Discussion

“To our knowledge this is the first field study providing evidence that ecological effects of agricultural pesticides can pose a serious risk to human health. Laboratory studies have already shown how pesticide pollution can increase risk of schistosomiasis^27^. We demonstrate a second mechanism with both field and laboratory data showing how such conditions favor the disease by benefitting the host snail. Host snails of schistosomiasis showed highest tolerance to insecticides amongst all tested macroinvertebrate taxa. Host snails were also solely found in habitats that were at least moderately affected by pesticides and eutrophication. In these conditions, snails replaced more sensitive potential competitor species. The study thus sheds light on an important risk factor for the transmission of schistosomiasis that has been largely overlooked in previous research on the ecology of host snails and in public health programs.

### Pesticide pollution in the study area

Concentrations of the highest exposure (TU_max_) ranged from <−5 to −1.21, representing a 10^th^ to <100,000^th^ of the acute lethal median concentration for standard test organisms. This range of pesticide exposure is comparable to several previous studies conducted in agricultural streams of Europe and Australia^22,30,35^.

Given the predominance of subsistence farming in the study area, the results illustrate that pesticide pollution in freshwater is not limited to intensified agriculture. Apparently, the risk of pesticide runoff from agricultural fields is high in the study region. During the rainy seasons in April - June and in October - December, heavy rainfalls erode the cleared land, as indicated by the high turbidity observed in streams during sampling. Because the water bodies are typically not protected by riparian strips from surface run-off, pesticides are washed from flooded agricultural fields into the streams and reservoirs^36^. In addition, plant protection products are sold in Kenya at comparably low prices without the need for a certificate of competence. This makes pesticides available even for small farmers who may lack enough training and equipment to comply to the proposed environmentally safe use.

The study focused on a broad set of agricultural pesticides typically detected in water. It cannot be excluded that the overall pesticide toxicity might have been even higher due to additional compounds such as pyrethroid insecticides that require different analytical methods. However, pyrethroids typically occur concurrently with the compounds detected and show a similar range of toxicity^35,37^. In previous studies^22,30,35^ pesticides have been sampled during the peak exposure following run-off events after heavy rainfalls. Event-triggered sampling was not feasible in our study area. However, we sampled during the main rainy season at which we expected the highest pesticide exposure from run-off. Therefore, the TU_max_ values determined to characterize the toxic pressure in this study are comparable to those of previous investigations.

### Effects of pesticides on schistosomiasis infection

Host snails of human-pathogenic schistosomes were found exclusively in freshwaters that were at least moderately polluted with pesticides (TU_max_ >= −3) and at least mesotrophic. Physicochemical and land-use parameters had no significant effect on the abundance of host snails, and additional pollutants such as pharmaceuticals, personal care products and industrial chemicals have been shown to cause considerably lower environmental risk than pesticides at the study sites^38^. This was observed across various habitats ranging from reservoirs to irrigation channels and streams.

The results support our hypothesis that agricultural pesticide pollution in tropical freshwaters increases the risk of infection with schistosomiasis: Snails as intermediate hosts of human-pathogenic schistosomes are mandatory to close the infection cycle, and humans can become infected only from larval forms (cercariae) released by snails into the water^6^. Besides freshwater contamination with infected human excreta and human contact with freshwater infested with cercariae, presence of host snails is a major risk factor for transmission^39^. Two human-pathogenic trematodes occur in the study region: *Schistosoma mansoni* parasitizes snails of the genus *Biomphalaria sp*. and causes intestinal schistosomiasis, whereas *S*. *haematobium* parasitizes certain snails of the *Bulinus africanus* complex and causes urogenital schistosomiasis^40^. Access to sanitation is often insufficient in the densely populated study area^11,41^, and therefore many people are exposed to non-sanitized freshwater during activities such as bathing (particularly school children), washing and field work^39^. This is especially pronounced at the shore of Lake Victoria which suffers from a high disease burden^11,42^. When traveling, schistosomiasis transmission may be imported to inland areas if host snails are present^43^. In these conditions, we expect that the risk of infection is influenced by the occurrence of intermediate host snails.

Our finding of pesticide-induced shifts in the community composition towards more snails are in line with various studies that reported significant ecological effects of pesticides even in streams with low concentrations resembling a TU_max_ of −2 to −4. These effects include changes in the macroinvertebrate community composition towards more tolerant taxa^30^, reduced leaf litter breakdown^37^ and the development of pesticide resistance^35^. The results, however, contrast the common perception of the environmental risk of pesticides. For example, according to the European framework for the registration of plant protection products, environmental concentrations <1% of the acute LC_50_ of the most sensitive standard reference organism are generally considered safe^44^; this would resemble a TU_max_ up to −2. No such threshold concentrations have been defined in Kenya, but pesticides need to be considered environmentally safe by the national Pest Control Product Board before registration^45^. The present study shows that pesticides nevertheless affect the community composition of freshwater macroinvertebrates and that these ecological effects can have serious consequences for human health, hence the need for revision of acceptable regulatory concentrations.

### Ecological mechanisms supporting host snails

Given the very high pesticide tolerance of snails compared to the pesticide concentrations measured in the environment, a direct effect of pesticides on the observed host snails appears unlikely. Instead, pesticides may indirectly favor host snails through adverse effects on their antagonistic species such as predators and competitors. A recent mesocosm study showed that herbicides and insecticides can favor host snails of human-pathogenic schistosomes through effects on predators and the support of periphyton as food source for snails (through effects on antagonistic planktonic algae)^27^. However, the study did not collect field data, nor did it discuss potential effects on competitor species^27^. The observed effects on predators in the mesocosm study contrast our results, probably because pesticide concentrations were 3–4 orders of magnitude higher than those observed at our study sites. Such concentrations may affect even taxa such as predators that showed generally intermediate pesticide tolerance in our tests and in previous studies^46^. We observed that with increasing pesticide pollution snails replaced grazing insects that are known to compete with the more tolerant snails^16^ and are generally highly sensitive to insecticides and some fungicides^46,47^. In fact, pesticides have been shown to affect the survival and emergence of aquatic insects at concentrations down to 0.005% (4 orders of magnitude below) of their acute LC_50_^48,49^. Moreover, the calculation of toxic units for additional trophic levels revealed highest risk for insects and crustaceans compared to algae and vertebrates in our study sites^38^. Therefore, we focused on the toxicity of pesticides to invertebrates. Our results indicate that in the field, pesticides favor snails mainly through negative effects on more sensitive competitors. Pesticide pollution was closely related with turbidity, as both factors increase with rainfall induced flooding^23,50^. Nevertheless, these factors showed contrasting effects on the abundance of host snails (increased incidence vs. decreased population density). We hypothesize that flooding results in a short-term reduction of host snail populations due to increased flow velocity^51^, whereas pesticide exposure in the long-term facilitates the establishment of tolerant taxa such as snails^30,52^. This may explain the generally low population densities of host snails observed during the rainy season and the more obvious link of pesticide pollution with incidence than with population density.

## Conclusions

The present case study illustrates that serious consequences of agricultural pesticide pollution arises for public health, even at concentrations considered safe within the traditional risk assessment. Given that pesticide application – particularly in developing countries - is predicted to increase 2- to 5-fold from 2000 to 2050 to meet the food demand of a growing human population, freshwater pollution and its ecological effects will aggravate^53,54^. The results underline the urgent need for reassessing the environmental risk of low pesticide concentrations and for integrated disease management that includes a focus on the regulation and management of pesticides in areas where schistosomiasis is endemic or might be introduced due to potentially favorable ecological conditions.

## Materials and Methods

### Acute toxicity tests

We investigated acute insecticide sensitivity for all macroinvertebrate taxa that could be found in sufficient quantities in October/November 2018 from 6 sites in the study area. Site selection was based on host snail availability, high macroinvertebrate diversity, and low presumed pesticide pollution as indicated by buffer strips to minimize testing populations that may have developed pesticide resistance^35^. Test organisms were collected using sweep nets, standard pint dippers and snail catchers; they were sorted and identified to family level in the field. The organisms were placed in plastic lunch boxes filled with water from the sampling site and aerated using battery-operated air pumps. The containers were cooled in a portable fridge at 18 °C in order to prevent mortality during transport to the laboratory. The organisms were acclimatized to test conditions overnight. Tests were performed in a shaded screen-house with temperatures ranging from 20 to 33 °C. Commonly applied agricultural insecticides comprise three major classes with distinct modes of action: Organophosphates/carbamates, neonicotinoids and pyrethroids. If sufficient test organisms were available, we tested the acute sensitivity to one of the most toxic substances among the organophosphates (diazinon) and neonicotinoids (imidacloprid) measured at the study sites, respectively. To increase environmental realism, we applied local plant protection products containing the active substance and additional carriers that might have affected the toxicity.

Tests were performed according to the Rapid Test protocol for field-collected organisms^55^ with minor modifications. Imidacloprid was applied as a 70% wettable granule formulation (Loyalty, manufactured by Shandong United Pesticide Industry Co., Ltd. Jinan city, China) and diazinon was applied as a 60% emulsified liquid formulation (Diazol, repacked and distributed by Laibuta Chemicals Ltd). Fresh stock solutions were prepared a few hours before each test dissolving the formulations in a 1:1 mixture of bottled water and activated carbon filtered stream water. This mixture was a compromise to minimize adverse effects from water to which the organisms had not been adapted, and to minimize potential effects from residual toxicity and from dissolved solids in the stream water which can absorb pesticides. Stock solutions of 165. mg active substance/L were left to stir overnight in amber vials; no additional solvents were applied.

Test organisms were exposed to 6 test pesticide concentrations including a control. For each taxon test concentrations were selected from the following geometric series such that they covered the expected range of a partial response from <5% mortality in the lowest concentrations to >95% mortality in the highest concentration: 0.001; 0.004; 0.014; 0.055; 0.209; 0.792; 3.01; 11.4; 43.5, 165 mg/L.The ranges expected to result in a partial response were identified from data bases^32,56^ and previous studies^35^ for related taxa and substances. The tests were performed in 100 ml glass vessels containing up to 5 individuals of the same species (predators were kept individually to avoid cannibalism). The test medium was constantly aerated using aquarium pumps connected to glass pipettes via a silicone tube. Only the glass pipette had contact with the test solution and the air flow was controlled through a clamp on the silicone tube. After 24 h and = 48 h, mobile, immobilized and dead individuals were counted. Individuals were considered immobilized when no movement was observed within 10 s of undisturbed observation or after probing with a rod; fanning of gills was not considered movement.

### Field sampling

48 study sites located in Homa Bay, Kericho, Kisii, Kisumu, Migori and Nyamira in Western Kenya were investigated from September–November 2017. The aquatic habitats were chosen from areas characterised by different types of land use and crops grown, identified using aerial photos from Google Maps. We classified the sampling sites according to habitat types (major tributary, minor tributary, irrigation channel, oxbow lake, reservoir or rice field) and the surrounding dominant land use within 50–100 m (natural, agricultural, semi-urban, urban or industrial). Agricultural land use was classified by farm type, subsistence or irrigation schemes and crop type (maize, tea, sugarcane or rice).

Streams, rivers and oxbow lakes were sampled across a 50-metre section whereas dams and irrigation schemes were sampled at four sub-sites. The aquatic habitats were surveyed for the presence of submerged, emerging or floating vegetation as well as algal bloom and the percentage of detritus cover. Depth was measured at the bank at point of sampling using a metre rule or was indicated as >1 m. Flow velocity was estimated with the drift approach. Additionally, we measured physicochemical parameters (temperature, conductivity, pH, dissolved oxygen, carbonate hardness, ammonium, phosphate, nitrate, nitrite and nitrite) and the turbidity on site using cholorimetric test kits (MACHEREY-NAGEL Quantofix, Düren, Germany), a turbidimeter (WTW TURB 355 IR, Weilheim, Germany), a multi-measurement probe (EXTECH ExStick EC500, Boston, USA) and an oxygen probe (EXTECH ExStick DO600, Boston, USA).

For pesticide analyses, grab samples were taken using pre-cleaned glass beakers. Briefly, oven dried 500 mL beakers were rinsed three times with the sample water and filled up to the top. After suspended solids settled, 1 mL aliquots were taken into five 2 mL autosampler amber glass vials (Phenomenex, Germany) using a volumetric pipette. All samples were immediately stored in a portable freezer (Waeco Compressor Cooler Box – 50 litres #CF-50) at −4 °C and transferred to the laboratory where they were stored at −20 °C until analysis. For quality control, sampling and trip blanks were taken during each sampling campaign.

Macroinvertebrates were sampled along four equal sections of the water body. Banks were sampled in a criss-cross fashion along the sampling points using littoral sweep nets, dippers and snail catchers; a standardised sampling procedure was predetermined to collect macroinvertebrates comprehensively within the different microhabitats and habitat types. In brief, each site was sampled for 30 minutes by two persons in parallel. Collected macroinvertebrates were sorted and counted in white plastic trays and preserved in 70% ethanol. Some host snails were transported to the laboratory and checked for *Schistosoma* infection. The snails were kept individually in a 24-well plate (Nunc 142475 Nunclon) and exposed to sunlight for a minimum of 30 minutes, and shed cercariae were = identified under a dissecting microscope (Zeiss AxioCam5 100–400x) and an identification key for cercariae^57^. Macroinvertebrates were identified under a dissecting microscope (Zeiss AxioCam5 100–400x) to the lowest taxonomic level possible with the available identification keys^58–67^. Based on these data we calculated the following biological indices: the overall macroinvertebrate individual number, the species richness, Pielou’s species evenness, the Shannon index for species diversity, the ASPT indicator for stream health from the South African Scoring System SASS^68^, and the dominance (relative abundance) of potential predator and competitor species of the host snails. We considered all taxa as potential predators that comprise more than a marginal proportion of predatory species in the study region that may feed on freshwater snails or their eggs. Similarly, we considered all taxa as potential competitors that comprise more than a marginal proportion of periphyton feeders or herbivores in the study area (Table S6).

### Pesticide analysis

Details on the analysis of pesticide residues in water samples have been described in Kandie *et al*.^38^. In brief, 25 μL of an internal standard solution containing 40 isotope-labelled compounds (40 ng/mL9, 25 µL of methanol and 10 μL of 2MNH4-formate buffer (pH 3.5) was added to each sample prior to instrumental analysis. Analysis was performed using high-performance liquid chromatography (HPLC, Ultimate 3000 LC) coupled to high resolution mass spectrometry (HRMS, QExactive Plus MS) from Thermo Scientific. The sample (100 µL) was directly injected for chromatographic separation (Phenomenex Kinetex c18 EVO, 50 × 2.1 mm, 2.6 µm particle size), equipped with a pre-column (5.0 × 2.1 mm) and 0.2 µm in-line filter using a methanol/water gradient containing 0.1% formic acid. Heated electrospray ionisation (ESI) was performed for both the negative and positive modes with combined full scan run (100–1500 m/z) at a nominal resolving power of 70,000 (referenced to m/z 200) and data-independent MS/MS fragmentation (DIA) at a nominal resolving power of 35,000. An isolation mass window of m/z = 50 (m/z range 122–860) or m/z = 260 (m/z range 860–1370) was used in DIA analysis. Matrix matched calibration standards were prepared for 11 calibration levels (ranging from 1 to 2,000 ng/L) using 1 mL filtered water from a pristine reference stream (Wormsgraben, Harz Mountains, Germany). Quantification of detected pesticides was performed using isotope–labeled internal standards of compounds with closest retention time to the target compound. Data evaluation was performed using MZmine (Version 2.38 http://mzmine.github.io/) and trace finder (Thermo, version 4.1 https://www.thermofisher.com/ke/en/home/industrial/mass-spectrometry/liquid-chromatography-mass-spectrometry-lc-ms/lc-ms-software/lc-ms-data-acquisition-software/tracefinder-software.html).

### Data analysis

All data were analyzed using the software R 3.5.2^69^. From the mortality observed in the acute toxicity tests we calculated the acute lethal median concentrations after exposure for 24 h (LC50_24h_) with 4-parameter log-logistic non-linear regression available with the package drc 3.0–1^70^. The parameters for the upper and lower boundary were fixed to 1 and 0, respectively. If a taxon had been tested at more than one date or from more than one site, data were merged prior to the analysis. Tests which showed >30% control mortality were excluded from analyses. The resulting LC_50_values were ranked in ascending order to obtain the species sensitivity distribution (SSD). This increase in the proportion of affected taxa with pesticide concentration was described using a quasibinomial generalized linear model (GLM) with a logit link function.

For all GLMs in this publication, *p*-values were obtained from likelihood-ratio tests that compared each model to a null model without the environmental variable. Depicted means and 95% confidence intervals were extracted from (generalized) linear models using the package effects 4.1-0^71^. Normally distributed residuals and homoscedasticity were confirmed using normal Q-Q plots and plotting residuals vs. fitted values; GLMs were inspected using scaled residuals available with the package DHARMa 0.2.0^72^. The effects of each environmental variable measured on the incidence of host snails were analyzed using one-way binomial GLMs (binary regression) with a complementary log-log link function which allows for a non-symmetric dose-response curve. The effects of each environmental variable measured on the density of existing host snail populations were analyzed using one-way GLMs with a zero-truncated negative binomial distribution of residuals and a log link function available with the package VGAM 1.0-6^73^. This way we dealt with overdispersion and with the missing possibility for the population density to be zero. Because many effects on the population density of host snails were driven by a single site (site 39) with extraordinarily high numbers of host snails and other macroinvertebrates, we repeated the analysis on the effects of host snail density with that site excluded. Only those environmental variables were considered in further analyses which significantly (*p* < 0.05) explained the population density after site 39 had been excluded.

Environmental variables that explained the incidence or population density of host snails were combined in a hurdle model available with the package pscl 1.5.2^74^. Hurdle models consist of two connected generalized linear models to simultaneously fit the incidence (zero part) and the population density (count part). Prior to modeling, the environmental variables were standardized (normalized and centered) to make the model parameters comparable. Environmental variables that significantly (*p* < 0.05) explained the incidence were incorporated in the zero part of the model (binomial GLM with complementary loglog link), and environmental variables that explained the population density were incorporated in the count part (zero-truncated negative binomial GLM with log link). To avoid overfitting, we applied an additive model without interactions. Then we sequentially removed all non-significant environmental variables based on a likelihood ratio test (backward elimination). Each time a variable had been removed, we started testing again with the least-significant of the remaining variables according to the model statistics.

Additionally, we fitted a hurdle model with all the environmental variables that on their own significantly explained the incidence or the population density included in both the zero and the count part. Hurdle models consist of two connected generalized linear models to simultaneously fit the incidence (zero part) and the population density (count part)^74^. We removed all non-significant effects from this full model in a stepwise backward-elimination process and then sorted the remaining effects based on the magnitude of their regression coefficients. Due to multicollinearity, selecting a single minimum adequate model can lead to different results depending on the method of model selection^75^. Therefore, this model was subjected to hierarchical partitioning. Because this procedure is currently not available for hurdle models in R, we extended the code of the function hier.part from the package hier.part 1.0–4^76^. The modified function started with a null model and each step included an environmental variable to both the zero and the count part at the same time. The improvement of the goodness-of-fit that resulted from the inclusion of an environmental variable was quantified using the log-likelihood.

Relations among the environmental explanatory variables were visualized using a principal component analysis (PCA) available with the function prcomp in basic R. A PCA reduces complexity by combining correlated environmental variables to few “supervariables” called principal components. The data were standardized prior to the analysis. Additionally, the association of the second principal component with the log-transformed number of macroinvertebrate individuals was analyzed using ordinary one-way linear regression.

The effect of pesticide pollution (TU_max_) on the overall community composition consisting of grazers, potential predators and other macroinvertebrates was analyzed using a permutational multivariate analysis of variance (PERMANOVA) available with the package vegan 2.5–4^77^. We also investigated the effect of pesticide pollution on the taxonomic composition of potential predators using a PERMANOVA. To investigate effects of the species composition of potential predators on the grazer composition, we performed a PCA on the predator composition. The first to fifth principal component was then fitted vs. the dominance of snails within grazers using quasi-binomial GLMs with a logit link function to account for the possibility of overdispersion. The proportions were weighted with the numbers of observed grazers. Similarly, the effects of pesticide pollution on the dominance of all grazers, of potential predators, and of snails within the guild of grazers were analyzed using quasi-binomial GLMs with a logit link function. The proportions were weighted with the numbers of observed individuals or of observed grazers, respectively. All data were analyzed using the software R 3.5.2^69^.

## Supplementary information

Supplementary Information.
