# Author Correction: Pesticide pollution in freshwater paves the way for schistosomiasis transmission

**DOI:** 10.1038/s41598-020-62951-7

**Published:** 2020-04-07

**Authors:** Jeremias M. Becker, Akbar A. Ganatra, Faith Kandie, Lina Mühlbauer, Jörg Ahlheim, Werner Brack, Baldwyn Torto, Eric L. Agola, Francis Mcodimba, Henner Hollert, Ulrike Fillinger, Matthias Liess

**Affiliations:** 1 Helmholtz Centre for Environmental Research − UFZ, Department System-Ecotoxicology, Permoserstrasse 15, 04318 Leipzig, Germany; 2 RWTH Aachen University, Department of Ecosystem Analysis, Institute for Environmental Research, Worringerweg 1, 52074 Aachen, Germany; 3 International Centre of Insect Physiology and Ecology (icipe), Human Health department, P.O. Box 30772-00100, Nairobi, Kenya; 4 Egerton University, Biological sciences, P.O Box 536-20115, Njoro, Kenya; 5 Ruprecht-Karl-University of Heidelberg, Faculty of Biosciences, Im Neuenheimer Feld 234, 69120 Heidelberg, Germany; 6 Centre for Biotechnology Research and Development, Kenya Medical Research institute (KEMRI), P.O. Box 54840-00200, Nairobi, Kenya; 7 The Technical University of Kenya, P.O. Box 52428-00200, Nairobi, Kenya; 8 Department Evolutionary Ecology and Environmental Toxicology, Institute of Ecology, Evolution and Diversity, Faculty Biological Sciences, Goethe University Frankfurt, Frankfurt, 60438 Germany

Correction to: *Scientific Reports* 10.1038/s41598-020-60654-7, published online 27 February 2020

The original version of this Article contained an error in Figure 1 where the two maps were overlapping. This has now been corrected in the PDF and HTML versions of the paper.

