# Supplementary material for: Pesticide pollution in freshwater paves the way for schistosomiasis transmission
Source: Sci Rep. 2020 Feb 27;10:3650. doi: 10.1038/s41598-020-60654-7 (PMC7046736; doi:10.1038/s41598-020-60654-7)
Supplement: Supplementary file 1 — Supplementary Information. [file 41598_2020_60654_MOESM1_ESM.pdf]

Supplementary Information

Pesticide pollution in freshwater paves the way for schistosomiasis transmission

Jeremias M. Becker[1,2], **Akbar A. Ganatra**[3,4*], Faith Kandie[2,3], Lina Mühlbauer[1,5], Jörg Ahlheim[1], Werner Brack[1,2], Baldwyn Torto[3], Eric L. Agola[6,7], Francis McOdimba[3,4], Henner Hollert [2,8], Ulrike Fillinger[3*], Matthias Liess[1,2*]

Shared first authorship of Becker JM and Ganatra AA

[1]Helmholtz Centre for Environmental Research – UFZ, Department System-Ecotoxicology, Permoserstrasse 15, 04318 Leipzig, Germany

[2]RWTH Aachen University, Department of Ecosystem Analysis, Institute for Environmental Research, Worringerweg 1, 52074 Aachen, Germany

[3]International Centre of Insect Physiology and Ecology (*icipe*), Human Health department, 00100 Nairobi, Kenya

[4]Egerton University, Biological sciences, P.O Box 536 Egerton, Kenya, 20115

[5]Ruprecht-Karl-University of Heidelberg, Faculty of Biosciences, Im Neuenheimer Feld 234, 69120 Heidelberg, Germany

[6]Centre for Biotechnology Research and Development, Kenya Medical Research institute (KEMRI), P.O. Box 54840-00200, Nairobi

[7]The Technical University of Kenya P.O. Box 52428 - 00200 Nairobi, Kenya

[8]Department Evolutionary Ecology and Environmental Toxicology, Institute of Ecology, Evolution and Diversity, Faculty Biological Sciences, Goethe University Frankfurt, Frankfurt 60438, Germany

*Corresponding authors. E-mail address: aganatra@icipe.org; matthias.liess@ufz.de; ufillinger@icipe.org

Table S1: Pesticides analyzed in the water samples. The acute LC_{50}values for *Daphnia magna*, *Chironomus sp.*, *Chironomus riparius* and *Hyalella azteca* were obtained from the PPDB and the ECOTOX data base[32,58]. In the PPDB data base LC_{50}values for 48 h exposure on *Chironomus* and *D. magna* were compared and the lowest value was used. If no values were available or if the tolerance of *D. magna* was exceptionally high (neonicotinoids), LC_{50}values for the same species or *H. azteca* were searched in the ECOTOX data base and the median value was used.

Name	CAS Nr.	Application	Class	LC_{50} [µg/L]	Test species	Duration	Source
2,4-Dichlorophenox. acid	94-75-7	Herbicide	Auxine	11,020.00	*C. sp.*	48 h	ECOTOX
Acetamiprid	135410-20-7	Insecticide	Neonicotinoid	11.56	*C. riparius*	96 h	PPDB
Ametryn	834-12-8	Herbicide	Triazine	28,000.00	*D. magna*	48 h	PPDB
Atrazine	1912-24-9	Herbicide	Triazine	85,000.00	*D. magna*	48 h	PPDB
Azoxystrobin	131860-33-8	Fungicide	Strobilurine	230.00	*D. magna*	48 h	PPDB
Bendiocarb	22781-23-3	Insecticide	Carbamate	30.00	*D. magna*	48 h	PPDB
Carbendazim	10605-21-7	Fungicide	Carbamate	150.00	*D. magna*	48 h	PPDB
Chlormequat	999-81-5	Herbicide	Growth regulator	31,700.00	*D. magna*	48 h	PPDB
Chlorothalonil-4-hydroxy	28343-61-5	Fungicide	Chloronitrile	76,000.00	*C. riparius*	48 h	PPDB
Desethylatrazine	6190-65-4	Metabolite	Triazine	5,100.00	*H. azteca*	96 h	ECOTOX
Desisopropylatrazine	1007-28-9	Metabolite	Triazine	7,200.00	*H. azteca*	96 h	ECOTOX
Diazinon	333-41-5	Insecticide	Organophosphate	1.00	*D. magna*	48 h	PPDB
Diethyltoluamid (DEET)	134-62-3	Repellent	Methylbenzamide	75,000.00	*D. magna*	48 h	PPDB
Diuron	330-54-1	Herbicide	Phenylurea	5,700.00	*D. magna*	48 h	PPDB

Dodemorph	1593-77-7	Fungicide	Morpholine	3,340.00	*D. magna*	48 h	PPDB
Ethofumesate	26225-79-6	Herbicide	Benzofuran	13,520.00	*D. magna*	48 h	PPDB
Fenuron	101-42-8	Herbicide	Acetylurea	502,000.00	*D. magna*	48 h	PPDB
Hexazinone	51235-04-2	Herbicide	Triazine	85,000.00	*D. magna*	48 h	PPDB
Icaridin	119515-38-7	Repellent		100,000.00	*D. magna*	48 h	PPDB
Imidacloprid	138261-41-3	Insecticide	Neonicotinoid	55.00	*C. sp.*	96 h	PPDB
MCPA	94-74-6	Herbicide	Auxine	190,000.00	*D. magna*	48 h	PPDB
Mepiquat	15302-91-7	Herbicide	Piperidine	68,500.00	*D. magna*	48 h	PPDB
Metalaxyl	57837-19-1	Fungicide	Acetylalanine	3,470	*D. magna*	48 h	PPDB
Metolachlor	51218-45-2	Herbicide	Chloroacetanilide	23,500.00	*D. magna*	48 h	PPDB
Metribuzin	21087-64-9	Herbicide	Triazinone	49,000.00	*D. magna*	48 h	PPDB
Pirimiphos-methyl	29232-93-7	Insecticide	Organophosphate	0.21	*D. magna*	48 h	PPDB
Simazine	122-34-9	Herbicide	Triazine	1,100.00	*D. magna*	48 h	PPDB
Terbuthylazine	5915-41-3	Herbicide	Chlorotriazine	21,200.00	*D. magna*	48 h	PPDB

Table S2: Pesticide pollution in water samples of the study sites. For each site, the maximum (TU_{max}) and the summed up (TU_{sum}) toxic unit of all analyzed substances is shown, together with the number of substances that could be detected and quantified. At sites 37 and 48 concentrations were too low to for quantification so that no TU_{max} and TU_{sum} could be calculated.

Study site	Coordinates	TU_{max}	TU_{sum}	Most toxic substance	Nr. detected	Nr. quantified
1	0°34'43.43"S; 34°36'35.40"E	-3.17	-3.17	Bendiocarb	14	6
2	0°34'53.96"S; 34°32'0.10"E	-4.44	-4.36	Simazine	15	4
3	0°28'29.67"S; 34°32'56.95"E	-2.04	-1.89	Bendiocarb	22	10
4	0°22'48.01"S; 34°38'30.92"E	-1.66	-1.65	Bendiocarb	16	6
5	0°40'50.77"S; 34°32'39.07"E	-2.30	-2.09	Bendiocarb	24	9
6	0°49'46.82"S; 34°23'44.12"E	-2.59	-2.39	Diazinon	17	8
7	0° 8'17.02"S; 34°56'8.82"E	-2.70	-2.63	Diazinon	23	12
8	0° 8'38.67"S; 34°58'20.86"E	-1.86	-1.85	Pirimiphos-meth.	24	12
9	0°10'18.07"S; 34°54'28.44"E	-1.86	-1.63	Pirimiphos-meth.	28	16
10	0°48'19.63"S; 34°41'54.93"E	-1.21	-1.06	Pirimiphos-meth.	27	17
11	0°22'38.69"S; 34°38'6.98"E	-1.90	-1.89	Bendiocarb	20	10
12	0°23'17.85"S; 34°38'30.02"E	-2.38	-2.36	Bendiocarb	21	10
13	0°33'40.22"S; 34°18'9.70"E	-3.85	-3.85	Bendiocarb	16	4
14	0°29'9.67"S; 34°31'2.85"E	-2.14	-2.11	Carbendazim	12	6
15	0°28'34.93"S; 34°32'41.36"E	-2.00	-1.98	Bendiocarb	28	10
16	0°27'5.24"S; 35°7'15.27"E	-2.60	-2.59	Bendiocarb	18	4
17	0° 9'0.91"S; 34°55'49.30"E	-2.58	-2.24	Carbendazim	18	9
18	0° 6'46.67"S; 34°47'29.77"E	-2.54	-2.53	Diazinon	15	4
19	0°28'38.92"S; 34°32'38.87"E	-2.25	-2.09	Bendiocarb	17	8
20	0°48'2.29"S; 34°43'45.10"E	-2.34	-2.34	Diazinon	14	1
21	0°32'20.58"S; 35°2'0.91"E	-2.21	-1.92	Diazinon	16	4
22	0°27'45.4"S; 34°33'55.1"E	-2.26	-2.25	Diazinon	20	9
23	0°59'50.59"S; 34°16'55.57"E	-2.36	-2.34	Diazinon	15	7
24	0°19'20.39"S; 34°47'20.33"E	-3.07	-3.06	Bendiocarb	13	4

25	0°53'54.15"S; 34°31'24.54"E	-2.46	-2.39	Diazinon	23	11
26	0°54'29.02"S; 34°33'28.89"E	-1.79	-1.67	Pirimiphos-meth.	18	10
27	0°28'34.33"S; 34°31'56.51"E	-2.07	-1.83	Pirimiphos-meth.	21	12
28	1° 3'53.69"S; 34°28'5.52"E	-2.33	-2.30	Diazinon	15	6
29	0°48'31.95"S; 34°43'58.93"E	-2.17	-2.10	Diazinon	19	9
30	0°53'8.15"S; 34°31'20.35"E	-2.29	-2.27	Bendiocarb	14	4
31	0°27'34.68"S; 34°35'40.87"E	-3.50	-3.43	Carbendazim	14	5
32	1° 1'18.24"S; 34°37'27.29"E	-2.40	-2.39	Diazinon	17	4
33	0°49'50.42"S; 34°44'44.98"E	-4.09	-3.88	Imidacloprid	19	8
34	0°49'4.79"S; 34°23'34.97"E	-2.55	-2.45	Bendiocarb	21	10
35	0°27'47.58"S; 34°32'55.31"E	-1.89	-1.88	Bendiocarb	20	10
36	0°46'39.89"S; 34°12'21.19"E	-6.44	-6.34	Diethyltoluamid	4	2
37	0°48'47.38"S; 34°13'15.06"E	/	/	Ametryn	6	0
38	0°39'27.61"S; 34°42'37.10"E	-2.26	-2.06	Bendiocarb	16	8
39	0°39'24.17"S; 34°41'57.84"E	-2.06	-1.98	Diazinon	17	10
40	0°30'48.53"S; 34°17'29.08"E	-5.81	-5.80	Simazine	8	2
41	0°23'35.22"S; 35° 0'35.88"E	-4.94	-4.94	Simazine	8	1
42	0°59'40.04"S; 34°17'26.47"E	-3.66	-3.66	Bendiocarb	9	2
43	0°59'40.04"S; 34°17'26.47"E	-1.81	-1.76	Diazinon	23	15
44	0°59'9.30"S; 34°35'5.25"E	-2.54	-2.53	Bendiocarb	20	10
45	0°27'5.46"S; 35°13'8.39"E	-3.93	-3.81	Imidacloprid	10	3
46	0°29'12.72"S; 35°10'58.85"E	-2.59	-2.24	Diazinon	17	9
47	0°30'54.82"S; 35° 4'49.80"E	-2.24	-2.23	Diazinon	12	3
48	0°19'1.31"S; 35° 0'22.66"E	/	/	Acetamiprid	1	0

Table S3: Ranking of the analyzed pesticides according to the environmental toxicity observed. The substances were ordered according to the maximum toxic unit (Max. TU) observed in water samples from all study sites. Additionally, the number of sites is reported at which a substance showed the highest toxic unit among all pesticides analyzed.

Pesticide	CAS Nr.	Application	Class	Max. TU	Most toxic at nr. of sites	Detected at nr. of sites
Pirimiphos-methyl	29232-93-7	Insecticide	Organophosphate	-1.21	5	6
Bendiocarb	22781-23-3	Insecticide	Carbamate	-1.66	17	31
Diazinon	333-41-5	Insecticide	Organophosphate	-1.71	15	36
Carbendazim	10605-21-7	Insecticide	Carbamate	-2.14	3	46
Acetamiprid	135410-20-7	Insecticide	Neonicotinoid	-2.80	0	39
Imidacloprid	138261-41-3	Insecticide	Neonicotinoid	-3.24	0	16
Simazine	122-34-9	Herbicide	Triazine	-3.83	3	46
2,4-Dichlorophenoxyacetic acid	94-75-7	Herbicide	Auxine	-4.32	0	48
Metalaxyl	57837-19-1	Fungicide	Acetylalanine	-4.40	0	47
Dodemorph	1593-77-7	Fungicide	Morpholine	-4.42	0	40
Ametryn	834-12-8	Herbicide	Triazine	-4.68	0	6
Hexazinone	51235-04-2	Herbicide	Triazine	-4.74	0	15
Atrazine	1912-24-9	Herbicide	Triazine	-4.88	0	45
Azoxystrobin	131860-33-8	Fungicide	Strobilurine	-4.98	0	32

Ethofumesate	26225-79-6	Herbicide	Benzofuran	-5.09	0	31
Metribuzin	21087-64-9	Herbicide	Triazinone	-5.42	0	8
Desethylatrazine	6190-65-4	Metabolite	Triazine	-5.61	0	47
Metolachlor	51218-45-2	Herbicide	Chloroacetanilide	-5.70	0	27
Diuron	330-54-1	Herbicide	Phenylurea	-5.76	0	15
Desisopropylatrazine	1007-28-9	Metabolite	Triazine	-5.77	0	17
Terbuthylazine	5915-41-3	Herbicide	Chlorotriazine	-5.96	0	31
Mepiquat	15302-91-7	Herbicide	Piperidine	-6.17	0	47
Icaridin	119515-38-7	Repellent		-6.17	0	47
Chlormequat	999-81-5	Herbicide	Growth regulator	-6.43	0	1
Diethyltoluamid (DEET)	134-62-3	Repellent	Methylbenzamide	-6.43	0	48
MCPA	94-74-6	Herbicide	Auxine	-7.15	0	48
Chlorothalonil-4-hydroxy	28343-61-5	Fungicide	Chloronitrile	-7.80	0	13
Fenuron	101-42-8	Herbicide	Acetylurea	-8.38	0	47

Table S4: Environmental effects on the incidence of schistosomiasis hosts in surface waters of the study area. Each environmental variable was fitted using a one-way binomial GLM with cloglog-link. The unit and the transformation of each environmental variable prior to analysis is given in squared brackets. For numerical variables, model coefficients are reported together with their standard error; *n* = 48 for each numeric model. For categorical variables, the back-transformed mean of each factor level is reported together with 95 % confidence intervals and the number of observations. χ^2 and *p* are reported from a likelihood ratio test against the null model without explanatory variables.

Numerical variable	Intercept	Slope	Res. df	X^2	*p*	
Flow velocity [ln(m/s)]	-1.64 ± 0.63	-0.02 ± 0.17	46	0.02	0.900	
Depth [ln(cm)]	-3.81 ± 2.65	0.57 ± 0.65	46	0.84	0.359	
Temperature [°C]	-1.50 ± 1.87	< -0.01 ± 0.07	46	< 0.01	0.969	
Conductivity [ln(µS/cm)]	-5.47 ± 3.07	0.79 ± 0.60	46	1.90	0.170	
Acidity [pH]	1.65 ± 2.85	-0.44 ± 0.39	46	1.02	0.312	
Dissolved oxygen [ln(mg/L)]	0.47 ± 0.70	-1.60 ± 0.58	46	8.06	0.004	**
Turbidity [ln(NTU)]	-0.92 ± 1.07	-0.13 ± 0.20	46	0.41	0.520	
Carbonate hardness [ln(°dH)]	-3.03 ± 1.16	0.89 ± 0.63	46	2.25	0.134	
NH_4 [mg/L]	-1.51 ± 0.38	-0.88 ± 2.69	46	0.11	0.746	
PO_4 [mg/L]	-1.24 ± 0.73	-0.02 ± 0.04	46	0.23	0.629	
NO_{3^-} [mg/L]	-1.13 ± 0.45	-0.06 ± 0.05	46	1.59	0.207	
NO_{2^-} [ln(mg/L)]	-0.72 ± 1.16	0.26 ± 0.35	46	0.54	0.463	
Species richness) [ln(*n* taxa)]	-5.29 ± 2.11	1.62 ± 0.86	46	4.39	0.036	*
Evenness [J’]	-2.07 ± 1.20	0.93 ± 2.14	46	0.16	0.688	
Species diversity [H’]	-4.21 ± 1.53	1.64 ± 0.84	46	4.42	0.035	*
SASS 5 [ln(ASPT)]	2.12 ± 3.11	-2.33 ± 1.99	46	1.56	0.211	
Macroinv. abund. [ln(*n* ind.)]	-2.47 ± 2.16	0.20 ± 0.48	46	0.18	0.672	
Dominance predators [%]	-2.05 ± 0.95	0.73 ± 1.31	46	0.33	0.564	
Dominance competitors [%]	-0.36 ± 0.45	-4.58 ± 1.87	46	9.09	0.003	**
Pesticide pollution [TU_{max}]	2.46 ± 1.69	1.72 ± 0.79	46	7.60	0.006	**
Emerged vegetation cover [%]	-1.37 ± 0.36	-1.73 ± 1.84	46	1.33	0.249	
Floating vegetation cover [%]	-1.61 ± 0.35	0.70 ± 1.91	46	0.13	0.716	
Submerged vegetation [%]	-1.69 ± 0.36	5.65 ± 3.95	46	1.26	0.262	
Detritus cover [%]	-1.57 ± 0.36	-0.03 ± 1.68	46	< 0.01	0.984	

Categorical variable		Mean (95 % CI)	*n*	Res. df	X^2	*p*
Habitat:	Main tributary	0.11 (0.03 – 0.38)	18	42	6.31	0.277
	Minor tributary	0.40 (0.17 – 0.74)	10			
	Irrigation channel	0.20 (0.03 – 0.79)	5			
	Oxbow lake	< 0.01 (0.00 – 1.00)	3			
	Reservoir	0.25 (0.07 – 0.68)	8			
	Rice field	< 0.01 (0.00 – 1.00)	4			
Land use:	Natural	0.30 (0.11 – 0.67)	10	44	6.37	0.095 ·
	Agricultural	0.16 (0.07 – 0.34)	32			
	Semi-urban	< 0.01 (0.00 – 1.00)	5			
	Industrial	1 (0.00 – 1.00)	1			
Farm type:	Natural	0.27 (0 .10– 0.63)	11	43	1.37	0.850
	Subsistence	0.21 (0.07 – 0.53)	14			
	Agroforestry	< 0.01 (0.00 – 1.00)	1			
	Commercial	0.13 (0.04 – 0.44)	15			
	Irrigation scheme	0.14 (0.02 – 0.67)	7			
Crop type:	Maize	0.15 (0.04 – 0.49)	13	35	3.93	0.415
	Rice	0.09 (0.01 – 0.49)	11			
	Sugar cane	0.42 (0.16 – 0.83)	7			
	Tea	0.14 (0.02 – 0.67)	7			
	Other	< 0.01 (0.00 – 1.00)	2			

Table S5: Environmental effects on the population density of schistosomiasis hosts in surface waters of the study area. Each environmental variable was fitted using a one-way GLM with a zero-truncated negative-binomial distribution and log-link. In the upper part of the table results are reported with all study sites considered where hosts snails had been found; $n = 9$ and res. df = 7 in all models. Only those environmental variables are presented that showed a (marginally) significant effect ($p < 0.1$); see Tab. S4 for a complete list of environmental variables tested and for the units of measurement. Below, effects of the same variables are shown when site 39 (with extraordinary mass development of host snails) was excluded as a highly influential outlier; here $n = 8$ and res. df = 6 in all reported models. Model coefficients are reported together with their standard error and McFadden's pseudo-R^2. χ^2 and p values are reported from a likelihood ratio test against the null model without explanatory variables

**Environmental variable**	**Intercept**	**Slope**	**Pseudo-R^2**	**X^2**	***p***	
			For all study sites			
Depth	11.66 ± 3.23	-2.26 ± 0.81	0.09	6.25	0.012	*
Acidity	10.37 ± 1.99	-1.09 ± 0.28	0.14	10.41	0.001	**
Turbidity	12.60 ± 2.95	-2.01 ± 0.60	0.07	5.00	0.025	*
PO_4	4.75 ± 1.09	-0.13 ± 0.06	0.04	2.79	0.095	·
Species richness	-5.98 ± 3.74	3.52 ± 1.49	0.06	4.47	0.034	*
Evenness	5.96 ± 1.33	-6.23 ± 2.41	0.06	4.02	0.045	*
Macroinvertebrate abundance	-3.92 ± 2.36	1.43 ± 0.50	0.11	7.90	0.005	**
Dominance predators	5.49 ± 0.84	-4.39 ± 1.21	0.13	9.08	0.003	**
Dominance competitors	1.52 ± 0.59	6.94 ± 2.68	0.09	6.22	0.013	*
			Without site 39			
Depth	2.93 ± 3.39	-0.19 ± 0.82	< 0.01	0.03	0.871	
Acidity	7.55 ± 6.72	-0.72 ± 0.90	0.01	0.72	0.395	
Turbidity	5.73 ± 1.43	-0.73 ± 0.29	0.09	4.50	0.034	*
PO_4	2.83 ± 0.58	-0.04 ± 0.03	0.03	1.73	0.188	
Species richness	-0.80 ± 2.54	1.22 ± 1.04	0.02	1.16	0.281	
Evenness	3.21 ± 0.86	-1.94 ± 1.50	0.02	1.07	0.302	
Macroinvertebrate abundance	-0.83 ± 1.83	0.68 ± 0.41	0.04	2.27	0.132	
Dominance predators	3.65 ± 1.02	-2.10 ± 1.37	0.05	2.33	0.127	
Dominance competitors	2.16 ± 0.51	0.02 ± 3.17	< 0.01	< 0.01	0.996	

Table S6: Classification of macroinvertebrate taxa in relation to the host snails of schistosomiasis.

Order	Family	Feeding type	Relation to snails
Ephemeroptera	Baetidae	Grazer	Competitor
Ephemeroptera	Caenidae	Grazer	Competitor
Ephemeroptera	Ephemeridae	Grazer	Competitor
Ephemeroptera	Heptageniidae	Grazer	Competitor
Ephemeroptera	Leptophlebiidae	Grazer	Competitor
Ephemeroptera	Oligoneuridae	Grazer	Competitor
Ephemeroptera	Polymitarcyidae	Grazer	Competitor
Ephemeroptera	Prosopistomatidae	Grazer	Competitor
Ephemeroptera	Teloganodidae	Grazer	Competitor
Plecoptera	Perlidae	Predator	Predator
Trichoptera	Ecnomidae	Predator, Grazer	Predator, Competitor
Trichoptera	Hydropsychidae	Filterer	Other
Trichoptera	Pisuliidae	Grazer	Competitor
Trichoptera	Polycentropodidae	Filterer	Other
Zygoptera	Chlorocyphidae	Predator	Predator
Zygoptera	Chlorolestidae	Predator	Predator
Zygoptera	Coenagriidae	Predator	Predator
Zygoptera	Lestidae	Predator	Predator
Anisoptera	Aeshnidae	Predator	Predator
Anisoptera	Corduliidae	Predator	Predator
Anisoptera	Gomphidae	Predator	Predator
Anisoptera	Libellulidae	Predator	Predator
Heteroptera	Belostomatidae	Predator	Predator
Heteroptera	Corixidae	Predator, Grazer	Predator, Competitor
Heteroptera	Gerridae	Predator	Predator
Heteroptera	Hydrometridae	Predator	Predator
Heteroptera	Naucoridae	Predator	Predator
Heteroptera	Nepidae	Predator	Predator
Heteroptera	Notonectidae	Predator	Predator
Heteroptera	Veliidae	Predator	Predator
Coleoptera	Dytiscidae	Predator	Predator
Coleoptera	Elmidae / Dryopidae	Grazer	Competitor
Coleoptera	Gyrinidae	Predator	Predator
Coleoptera	Haliplidae	Predator, Plant sucker	Predator, Other
Coleoptera	Hydrophilidae	Predator	Predator
Coleoptera	Noteridae	Predator	Predator
Coleoptera	Scirtidae	Predator	Predator
Diptera	Chironomidae	Filterer, Grazer	Other
Diptera	Culicidae	Filterer, Grazer	Competitor, Other
Diptera	Muscidae	Predator	Predator
Diptera	Simulidae	Filterer	Other
Diptera	Tipulidae	Shredder	Other
Lepidoptera	Pyralidae	Herbivor	Competitor
Crustacea	Atyidae	Grazer, Filterer	Competitor
Gastropoda	Ampulariidae	Grazer, Herbivor, Predator	Snail
Gastropoda	Ancylidae	Grazer	Snail
Gastropoda	Hydrobiidae	Grazer	Snail
Gastropoda	Lymnaeidae	Grazer, Herbivor	Snail
Gastropoda	Physidae	Grazer	Snail
Gastropoda	Planorbidae	Grazer, Herbivor	Snail
Gastropoda	Thiaridae	Grazer	Snail
Gastropoda	Viviparidae	Grazer	Snail
Annelida	Hirudinea	Predator	Predator
Annelida	Oligochaeta	Detritivor	Other
